# U-SplitDoRA: an improved privacy-preserved U-shaped split parameter-efficient fine-tuning framework through weight decomposition for large language models

**DOI:** 10.3389/frai.2026.1807960

**Published:** 2026-05-29

**Authors:** Samar Singh, Brindha Subburaj, R. Alagewaran, Sherly Alphonse

**Affiliations:** 1 School of Computer Science and Engineering, Vellore Institute of Technology, Chennai, India; 2 School of Computing, SASTRA Deemed University, Thanjavur, India

**Keywords:** distributed learning, large language models, parameter-efficient fine-tuning, split federated learning, weight-decomposed low-rank adaptation

## Abstract

As large language models (LLMs) are getting bigger with respect to the parameter count, ranging from a few million to billions, methods like parameter-efficient fine-tuning (PEFT) have emerged as a crucial approach for adapting these LLMs, such as GPT, Llama, and DeepSeek, to resource-constrained and privacy-sensitive environments. The robustness of large language models (LLMs) while operating on complex tasks and with large datasets makes them feasible for various application domains. This also demands the availability of more public datasets to train LLMs in the future. The federated learning (FL) technique, where several entities collaboratively train a machine learning model without sharing their data, is a widely adopted decentralized training framework. This is followed by a central server, which aggregates the models to create a global model. FL LLM fine-tuning has gained attention recently to overcome the aforementioned training data scarcity issue. LLMs are collaboratively fine-tuned by several data owners without disclosing their private data. The large number of trainable parameters has a direct effect on training such complex models on the client side. The split learning technique, through model partitioning, solves the training overhead by offloading certain training tasks to the server side. Previous research based on the split learning approach for FL LLM fine-tuning, namely SplitLoRA and HSpliLoRA, sets the foundation for further research in this direction. Frameworks like SplitLoRA have already enabled collaborative fine-tuning through model partitioning, but privacy preservation and adaptation quality remain open research challenges. U-SplitDoRA—an improved privacy preserved U-shaped split parameter-efficient fine-tuning framework through weight decomposition for large language models—is proposed. U-SplitDoRA harnesses the parallelization power of FL through the split learning approach, using weight-decomposed low-rank adaptation (DoRA) as the PEFT technique. To further address privacy concerns, the U-shaped paradigm is adapted while splitting the model. By partitioning the model into three parts (head, body, and tail), with the head and tail remaining on the client side while the body is on the server side, it ensures that neither raw data nor labels are exposed to the server, thus providing strong privacy. Additionally, replacing low-rank adaptation (LoRA) with DoRA as the PEFT method further enhances adaptation, as it updates both the magnitude and direction of weights, resulting in superior expressiveness and reducing the gap between PEFT fine-tuning and full parameter fine-tuning to a minimal margin. Experiments are conducted using GPT-2-S and GPT-2-M trained on the E2E benchmark dataset. The simulation results confirm that U-SplitDoRA attains better accuracy scores and convergence speed than other SOTA LLM fine-tuning frameworks. Thus, the proposed method addresses key gaps in privacy and adaptation quality, paving the way for efficient, robust, and privacy-preserving fine-tuning of LLM models in a distributed setting.

## 1 Introduction

Large language models (LLMs) are a major advancement in the field of Artificial Intelligence. LLMs enable machines to perform natural language processing tasks like text summarization, text generation, question answering, and language translation. Recently, LLMs have been adapted to tasks that include texts, images, audio, and video. LLMs are built on deep learning architecture, specifically originating from the transformer architecture (Vaswani et al., 2017). Google’s BERT (Devlin et al., 2019), Meta’s Llama (Touvron et al., 2023), and Deepseek R1 (DeepSeek-AI et al., 2025) are widely used models. LLMs are trained on large datasets to learn the context and semantics of the language well. LLMs are highly scalable and can be fine-tuned to perform specific tasks.

LLMs exhibit robust performance in varied domains such as healthcare (Meng et al., 2024; Vavekanand, 2026; Vavekanand et al., 2026), software development (Hou et al., 2024), and computer vision (Hamadi, 2023). Data exhaustion has become a critical issue, as that the availability of high-quality data is expected to reduce in the near future. This makes the training of data-hungry LLMs difficult (Villalobos et al., 2022). The surge in meeting the data requirements to train LLMs through model-generated data and combining available data further highlights the effects of the data scarcity issue.

The technical advancements in the field of sensors, Internet of Things (IoT) devices, and associated technologies have made it feasible to collect and aggregate large and distributed data. However, data privacy issues and communication barriers in transferring this data make data sharing difficult. AI firms like Google have the capability to collect huge volumes of private data and train LLMs using it. Med-PaLM (Singhal et al., 2025), a medical LLM for healthcare tasks, Codex (Mark, 2021) for code generation, and PaLM-E (Driess, 2023), a robotic model, are a few examples of LLMs. The downside of training LLMs independently on private data is that not all private data owners have sufficient volumes of data to train LLMs, which also incurs computation and communication overhead.

Collaborative training techniques like Federated Learning, where multiple organizations train LLMs without sharing their data, are important for harnessing the power of LLMs. Federated Learning (FL) is a machine learning technique where several private data owners or clients collaboratively train a machine learning model with private data in their local environments. The model updates, such as the gradients, are transferred to the server for aggregation to improve the global model. Extending the FL technique for LLM training/fine-tuning has recently been practiced to tackle the data scarcity problem (Ye, 2024; Cai et al., 2023).

FL LLM training/fine-tuning is a collaborative LLM training paradigm where multiple data owners train the LLMs on their private data within their environments, and the model parameters are sent to a central server, which aggregates them for global model updates (Koneˇcn’y et al., 2016; Zhang et al., 2024). The main limitation of this approach is that the client devices where the LLMs are locally trained are resource-constrained. LLMs with a large number of trainable parameters make the model size huge, making it difficult to train them using private data at the client end.

To meet the computational demands of LLMs, the split learning (SL) technique (Vepakomma et al., 2018) is adapted. SL is a distributed, privacy-preserving machine learning framework where the deep learning model is split and trained between the client and server. The major training workload is carried out on the server side. The model is partitioned accordingly, and the activations/gradients are exchanged among the client and server in the SL paradigm (Lin et al., 2024; Lyu et al., 2023).

**Figure 1:**
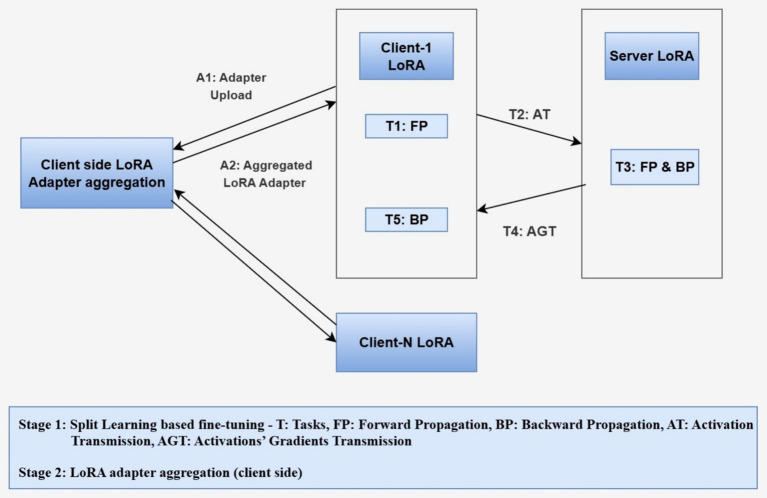
Overview of the split learning framework.

An illustration of the split learning-based LLM fine-tuning framework through PEFT is given in Figure 1. It combines the benefits of federated learning for parallelizing the training and split learning for model splitting and is integrated with the parameter-efficient fine-tuning technique (PEFT).

The SL LLM fine-tuning methods have recently been explored. In SplitLoRA (Lin et al., 2024), a split learning-based LLM fine-tuning approach using low-rank adaptation (LoRA) as PEFT is presented. The attained results are compared with other federated and centralized LLM fine-tuning frameworks; the results show the significance of split learning for LLM fine-tuning. In another SL LLM fine-tuning framework named HsplitLoRA (Lin et al., 2025), a heterogeneous split learning technique considering the heterogeneity of computing resources on the client side is presented. The decomposition rank of LoRA is chosen dynamically according to the computational capabilities of the client device. The accuracy and convergence results exhibit the robustness of the framework. A recent framework, SplitLLM (Zhang et al., 2025), proposes a hierarchical SL for LLM fine-tuning in a wireless network. The training is split between the end user and the edge server, while the aggregation is performed at the cloud server. The framework is suggested for wireless networks with a large number of users.

While the SL LLM fine-tuning paradigm seems promising, there are critical open challenges that need to be addressed. Firstly, the concern of privacy is often overlooked as there are multiple clients and a single centralized server. Unwanted data leaks or leaks of labels on the server side might render the real-time use of this approach ineffective, as users with sensitive information on these devices might refrain from using such methods and prefer the traditional approach to train their models, trading off the quality of predictions for privacy. Even though it is crucial in real-time to maintain this privacy, it remains a relatively unexplored field in the SL + PEFT paradigm. Second, there is an inherent loss of performance of the model in terms of accuracy, precision, and many other metrics like ROUGE and METEOR due to the usage of PEFT methods like LoRA. In some critical tasks, such as medical analysis tasks, this loss is unacceptable and further reduces the feasibility of the existing frameworks in the current paradigm.

To overcome these challenges, we propose a U-shaped PEFT framework built on split learning and a groundbreaking PEFT method named DoRA fine-tuning, referred to as U-SplitDoRA. First, to prevent any form of data leaks, we ensure that the initial layers where data is seen by the model and the final layers where predictions are made (or where labels are predicted) both reside on the client side, preventing any data leaks. The server still contains most of the layers, but as it only has the body layers, it is practically blind, as it can only compute activations and update gradients that originate from the client. Secondly, rather than using LoRA as the PEFT method, we opt to use DoRA (Liu et al., 2024), which shows better performance than LoRA and it is nearly identical to full parameter training, as it decomposes pre-trained weights into magnitude and direction. It employs LoRA for updating the directional component only, as training the magnitude might lead to a loss of valuable learned information. This approach closely resembles the learning capacity of full fine-tuning without additional inference overhead.

The major research contributions are as follows:

- To the best of our knowledge, U-SplitDoRA is the first LLM fine-tuning framework that integrates SL and PEFT with a heavy focus on privacy, enabling real-time applications of collaborative learning.
- We propose a U-shaped server–client architecture for split learning-based LLM fine-tuning, reducing data exposure through architectural design and encouraging users to share their data with improved trust, thereby increasing the performance of the models as they now have more data to train on.
- To ensure that we achieve near-identical performance to well as the full fine-tuning method, we incorporate DoRA adapters, which consistently demonstrate superior or competitive performance compared to LoRA across the evaluated settings.
- We empirically evaluate U-SplitDoRA through extensive experiments. The results demonstrate that U-SplitDoRA achieves competitive or superior performance compared to existing SOTA LLM fine-tuning frameworks across most evaluated metrics while providing benefits in data privacy inherent in the U-shaped structure introduced, while simultaneously decoupling magnitude and direction updates via DoRA, enabling faster convergence.

While SplitLoRA (Lin et al., 2024) and HsplitLoRA (Lin et al., 2025) establish the split learning + PEFT paradigm, they adopt a linear cut-layer architecture that exposes output logits to the server and rely on LoRA for parameter adaptation. U-SplitDoRA advances this direction in two orthogonal but complementary ways: It replaces linear partitioning with a U-shaped architecture that ensures the server never sees raw tokens or final prediction logits for privacy-sensitive deployments, and it replaces LoRA with DoRA, whose magnitude-direction decomposition removes the coupled update constraint that limits LoRA’s expressiveness. This combination has not been explored in prior literature.

The article is organized as follows: In section 2, the related works are discussed. The proposed methodology is detailed in section 3. The results and their discussion are presented in section 4. In section 5, the conclusion and scope for future work are discussed.

## 2 Related works

Recent research on large language models, split learning, parameter-efficient fine-tuning, and federated learning frameworks is discussed in this section.

### 2.1 Large language models

LLMs have gained much attention in recent years and are widely applied across several domains successfully. This is feasible due to the availability of robust deep learning models, the compute resources, and access to a large volume of training datasets. Major AI technology companies have developed and released their own LLMs. OpenAI’s GPT-4 (OpenAI, 2023), Google’s Gemini 1.5 (Gemini Team, 2024), Deepseek AI’s Deepseek-v3 (DeepSeek-AI and Liu, 2024), and Anthropic AI’s Claude 3 (Anthropic, 2023) are a few notable models.

LLMs were initially applied to various natural language processing tasks like text generation (Liang and Wang, 2024; Zhao et al., 2023), text summarization (Basyal and Sanghvi, 2023; Liu and Shi, 2025), language translation (Zhu and Liu, 2024; Huang and Liu, 2024), question answering (Yue, 2025), and text classification (Zhiqiang et al., 2024), and have demonstrated significant performance. Recently, LLMs have been extensively applied to various other tasks as well, such as in the healthcare sector for medical Q&A, diagnosis support, and medical image analysis. Google’s PaLM2 (Anil, 2023) is a pioneering model used for these tasks. In the finance sector, LLMs like BloombergGPT (Shijie and Ozan, 2023) are used for making investment decisions.

LLMs have now scaled up in size and have been trained using huge volumes and varieties of data, resulting in better generalization. Generalization is the ability of the model to perform well on new or unseen data. GPT-4 and Claude 3 have hundreds of billions of parameters and are trained on vast varieties of datasets. These features make them suitable for handling various tasks directly or through task-specific fine-tuning and have demonstrated superior performance.

### 2.2 Split learning

In the machine learning paradigm, a model exhibits robust performance when it is trained on more data, especially for complex tasks where the model needs to be trained on large and diverse datasets. This, however, comes with serious issues regarding data privacy. In healthcare, finance, and other sensitive applications, data owners may be reluctant to share their data due to privacy concerns. To address this situation, the federated learning (FL; McMahan et al., 2017) technique is adopted. FL is a machine learning approach where models are trained by data owners in their own environments in a decentralized manner without the need to share their data. The data owners/clients share model update parameters such as gradients with the server, where aggregation is carried out to improve the model; this updated model is then used by the clients for further training.

LLMs are growing bigger and better and need to be trained with more data; the availability of quality public datasets will reduce in the future. FL LLM training on distributed private data is an alternative to train the data-hungry models with large volumes of data to attain better performance. The size and number of trainable parameters in LLMs are large, which makes training or fine-tuning LLMs on the client side with their private raw data difficult, as it demands a heavy computational setup and also involves communication overhead. In many cases, the client is observed to be in a resource-constrained environment. Split learning (SL; Ryu et al., 2022) is a promising solution to the challenges in FL LLM fine-tuning tasks, such as resource constraints and data privacy issues. In SL, the model is partitioned between the client and server, and the primary training task is offloaded and carried out at the server. The activation and gradients are exchanged between the client and server entities instead of the model itself. The above SL method has gained much attention, and several research works have been conducted to further improve the distributed machine learning framework.

Several SL configurations have been presented recently (Duan et al., 2022), such as a simple vanilla configuration, where the model is partitioned at a designated point called the cut layer. The client entity trains the model up to this point, and the rest of the training is done at the server. The gradients are backpropagated up to this point at the server, and the gradients are sent back to the client to complete the backpropagation at the client end. Another representative SL technique is the U-shaped configuration, where neither raw data nor labels are shared with the server. The end layers of the model, which include input and output, are assigned at the client end, while the other layers are retained at the server, which improves the privacy factor by not exposing the labels. The other SL configuration is vertically partitioned data, which is adapted when clients have multi-modal data.

An efficient parallel split learning framework (Lin et al., 2024) is proposed for edge devices that are resource-constrained. The parallel split learning framework is improved by a key feature to reduce the overhead in communication and computation by aggregating the activation gradients of the last layer, which significantly reduces training at the server side. To address the data privacy concerns in the SL framework, the U-shaped parallel split learning (U-PSL) technique is presented (Lyu et al., 2023). In U-PSL, the SL framework is improved to preserve data privacy by eliminating the need to share raw data and labels. The model is split into head, body, and tail parts. The head and body, which involve the input and output layers of the model, are retained at the client end, and the rest of the layers are trained at the server end. This approach overcomes the privacy concerns related to sharing the labels. The performance result of the model is similar to that of other SL frameworks.

SL technique is used in various applications. For instance, in recent research, SL is used in 6G edge networks (Lin et al., 2024). The edge IoT devices are resource-constrained, and training deep learning models on such devices is practically difficult. To overcome this, the SL technique is used, and the major training workload is done on the server side. To adapt SL for edge networks, the architecture design is improved to accommodate model transfer, resource allocation, etc. A resource-efficient SL framework for resource management in the 6G network is presented. The split federated learning (SFL) approach (Thapa et al., 2022) is proposed to improve the computation time of the SL technique. SL, being a practical solution for training models in resource-constrained devices, also comes with the drawback that it is slower when relay-based training is practiced in a multi-client scenario. SFL harnesses the benefits of training models in parallel across multiple clients from FL and the benefits of partitioning the model from SL. SFL also aggregates the sub-models at the client and server for synchronizing the global model. Research on SFL for LLM training/fine-tuning is an emerging research field.

### 2.3 Parameter-efficient fine-tuning

The recent proliferation of LLMs has advanced their application to various NLP tasks and other domains as well. Fine-tuning these LLMs for a specific task demands fine-tuning all the model parameters. This full parameter fine-tuning is computationally expensive and also brings communication overhead in the SL framework. PEFT techniques are alternatives to reduce the above computational burden, in which the number of trainable parameters is significantly reduced. Several state-of-the-art PEFT techniques have been demonstrated to effectively fine-tune LLMs by training only a subset of parameters.

There are several classes of PEFT methods available in recent literature. The first type is adapter-based PEFT methods. Parameter-efficient transfer learning for NLP tasks is presented through adapter modules by including new layers in the original network with a lesser number of trainable parameters (Houlsby, 2019). This adapter-based fine-tuning attains results that are on par with respect to SOTA techniques. In contrast to the above, a unified view is presented (He et al., 2022), where the adapter modules are integrated with the original model layers for performance improvement, and the earlier sequential adapters are replaced here with parallel adapters. Adapter module-based PEFT techniques are further improved through shared hypernetworks (Mahabadi et al., 2021), which is a learning framework for multiple tasks. This approach is beneficial in two ways: hypernets allow knowledge sharing across tasks, and, further, task-specific adapters help the model for individual task adaptation.

The second class of PEFT methods is prompt-based, where prompts, which are the tokens included in the input, are learned during training. Conditioned frozen models through soft prompts are presented and demonstrated to be a robust PEFT technique when compared to other previous variants (Lester et al., 2021). The prompts are represented through random initialization or by selecting embeddings from the model’s vocabulary or the embeddings that list the output classes. In recent research, residual prompt tuning through residual reparameterization is presented (Razdaibiedina et al., 2023). Prompts are used in two ways: the regular way of embedding soft prompts and additionally included reparametrized prompts to make better decisions, giving more flexibility to the model. A non-intrusive variant of the PEFT technique for multimodal modeling through instruction tuning named AdaLink is presented (Wang, 2023). A total of just less than 0.02% of parameters are tuned, yet it exhibits competitive performance when compared to SOTA full fine-tuning techniques.

The third class of PEFT techniques is low-rank adaptation (LoRA; Hu et al., 2022). Using LoRA, the total number of trainable parameters is reduced, and it does not add additional inference burden. This is achieved by using two small matrices to estimate weight changes and fine-tuning them. Several improved variants of LoRA are proposed. Vector-based random matrix adaptation (VeRA) is presented, in which one pair of low-rank matrices is used and adapted using scaling vectors (Kopiczko et al., 2024). Another variant of LoRA, named Adaptive budget allocation for PEFT (ADALoRA), is suggested, where singular value decomposition is employed. In this method, singular values that are not significant are removed (Zhang, 2023). Through performance evaluations, it is reported that AdaLoRA works better than other techniques in low-budget settings.

FedPara, a communication-efficient parameterization technique for federated learning, is presented (Hyeon-Woo et al., 2022). In FedPara, the Hadamard product is used with low-rank weights which reduces the communication burden. In another recent work, weight-decomposed low-rank adaptation (DoRA) is presented (Liu et al., 2024). As the name implies, the weights are decomposed into magnitude and direction. These weights are then adapted separately; the performance of DoRA is closer to full fine-tuning, which shows learning superiority over LoRA.

### 2.4 Federated learning LLM PEFT techniques

In this section, research papers addressing LLM fine-tuning using PEFT techniques in a collaborative approach like federated learning are reviewed. An industrial-grade federated learning approach (FATE-LLM) is presented for PEFT of LLMs (Fan et al., 2023). LoRA and P-Tuning-V2 are used as PEFT techniques. The results of FATE-LLM are better than client-side fine-tuning and are worse when compared to centralized fine-tuning. In other work, Federated Scope LLM (FS-LLM; Kuang et al., 2023) is presented; FS-LLM is an end-to-end FL LLM fine-tuning framework, where several datasets are packaged together. The PEFT technique is used to reduce the trainable parameters and is optimized for parallelism. A federated intrusion tuning (FedIT) approach (Zhang et al., 2023) is presented by exploiting diverse instructions. GPT-4 is used for evaluation; LoRA-based PEFT is claimed to further improve the performance of FedIT.

Such LLM fine-tuning on the client side is highly challenging due to resource constraints. The split learning technique is a new addition to FL LLM fine-tuning models. The proposed research work addresses these challenges by adapting the split learning technique for LLM fine-tuning through a decentralized federated learning approach.

## 3 Methodology

This section provides an explanation of the proposed framework for privacy-preserving distributed large language model fine-tuning. Additionally, it includes a detailed description of the dataset used and the preprocessing steps applied to the data.

### 3.1 Proposed framework

**Figure 2:**
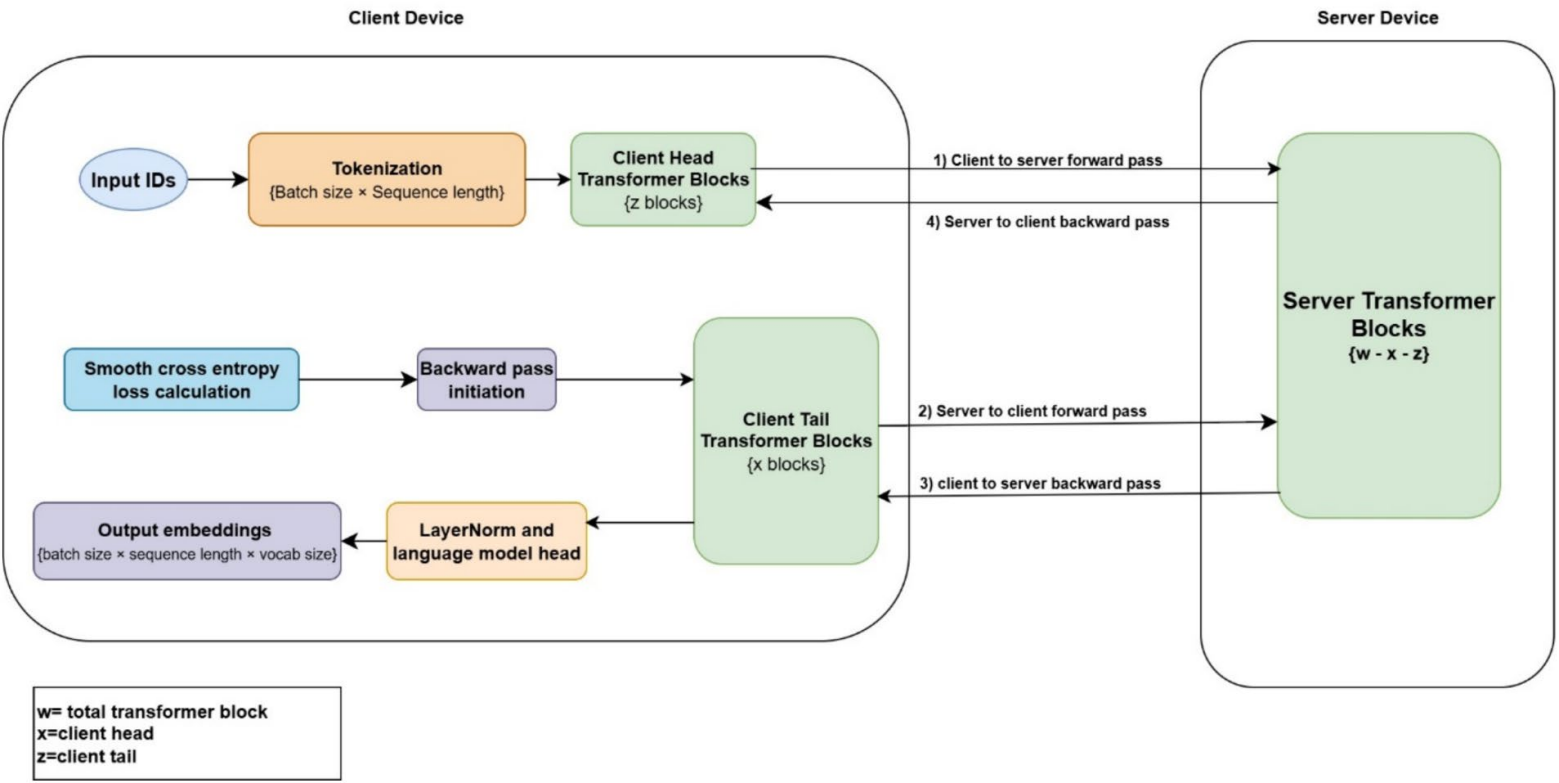
Workflow of the proposed U-SplitDoRA framework.

The proposed framework, U-SplitDoRA, aims to enable privacy-preserving distributed fine-tuning of large language models by employing a U-shaped split learning architecture combined with DoRA adapters. The architecture of U-SplitDoRA begins with partitioning the pre-trained GPT-2 model into three distinct components: client-side initial layers, server-side middle layers, and client-side final layers. This configuration ensures that raw input tokens and final output predictions never traverse network boundaries, providing a structural privacy advantage over linear-split architectures while maintaining model performance. The partitioned model leverages weight-decomposed low-rank adaptation (DoRA; Liu et al., 2024) instead of traditional LoRA, which decomposes weight updates into magnitude and directional components for more expressive, parameter-efficient fine-tuning. The framework processes the E2E NLG dataset through distributed training, where clients handle embedding layers and output generation while the server manages the intermediate transformer layers. The feature vectors obtained from the client-side initial processing are transmitted to the server for middle layer computation, then returned to the clients for final prediction and loss calculation. The detailed architecture diagram of the proposed framework is depicted in Figure 2.

The U-SplitDoRA framework includes DoRA adapters to decompose weight updates into magnitude and directional components, ensuring that the semantic dependencies and contextual relationships in the data are captured effectively. Different methods to facilitate the flow of gradients and activations between the client and server components are also employed. Each client consists of transformer layers that are wrapped under DoRA adapters, normalization layers, and communication protocols, which collectively work to extract knowledge from the data while still maintaining privacy. The input meaning representations and corresponding target texts are processed through their respective client-side DoRA adapters. The generated intermediate activations are transmitted to the server, processed through middle layers, and returned to clients for final text generation.

### 3.2 Dataset preparation

Over the years, centralized fine-tuning approaches have been the primary method for researchers to adapt large language models to specific downstream tasks. However, recent privacy concerns and computational constraints have led to increased interest in distributed learning paradigms that can preserve data privacy while maintaining model performance. Therefore, there is a need to explore frameworks that would provide substantial training efficiency while having a significant impact on model adaptation capabilities. One such approach is the E2E NLG dataset (Novikova et al., 2017), which provides structured meaning representation paired with natural language descriptions, enabling comprehensive evaluation of text generation capabilities.

**Figure 3:**
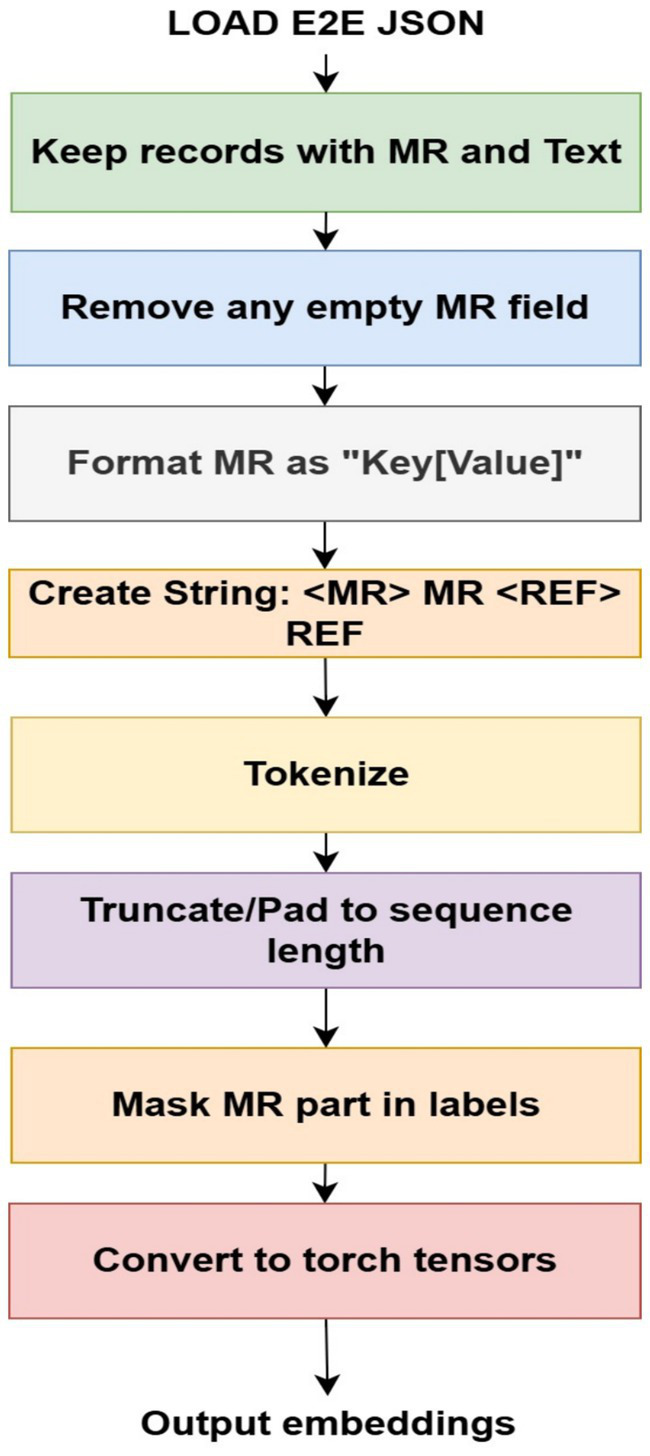
Workflow—dataset preparation.

For our study, we extracted data from the E2E NLG dataset, comprising meaning representations and corresponding target texts from the training, validation, and test splits. The extracted data is grouped into batches by consolidating all samples within each training iteration. Before this grouping, the raw data undergoes several preprocessing steps. The raw data extracted from the datasets consists of structured meaning representations in various formats and natural language targets. To handle the tokenization for both input meaning representations and output target texts, tokenization libraries are used, which ensure that the text sequences are suitable for model consumption. Additionally, all sequences are either padded or truncated to maintain consistent input lengths and standardize the data format. The meaning representations are combined with target texts to create input–output pairs suitable for autoregressive language modeling. Both the input and target sequences are normalized using appropriate tokenization schemes. For the input data, normalization involves converting representations to natural language prompts to ensure uniformity in input features. Target data is also put through the process of normalization, which ensures that all the text sequences are of the same sequence lengths, allowing the model to learn effectively across different sequence lengths. This process helps improve the overall robustness of the proposed framework. The steps in data preprocessing are given in Figure 3.

The text data is processed using GPT-2 tokenization, and the input sequences are formatted as “MR: [meaning_representation] Text:” followed by the target text. This process is clearly described in the section below. Each training example represents an independent meaning representation-to-text generation task, as given in Equations 1–3. This pipeline used to process the input dataset is illustrated in Algorithm 1.

ALGORITHM 1 Dataset preparation
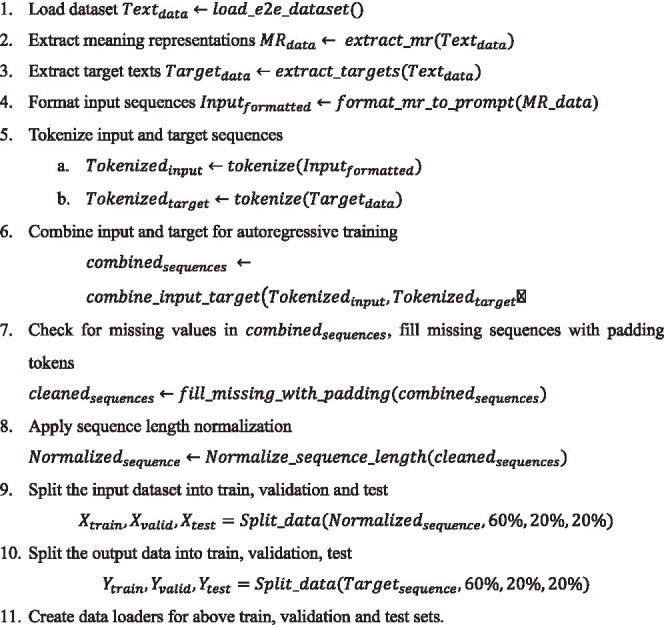

$$X(i)=\text{tokenize}(\mathrm{MR}:[\mathrm{MR}\_i]\;\text{Text}:)\;X(i)\in (1,\text{inpu}t_{\text{length}})$$ (1)

$$Y(i)=\text{tokenize}(\text{targe}t_{\mathrm{tex}t_{i}})\;Y(i)\in (1,\text{targe}t_{\text{length}})$$ (2)

$$S(i)=\text{concatenate}(X(i),Y(i))\;S(i)\in (1,\text{sequenc}e_{\text{length}})$$ (3)

where X(i) is the tokenized input prompt for the $i^{\mathrm{th}}$ meaning representation. Y(i) represents the tokenized target text for the $i^{\mathrm{th}}$ example. S(i) is the combined sequence for autoregressive training. Each example is processed independently without temporal dependencies, as each meaning representation–target pair constitutes a standalone text generation task.

The training inputs X(i) and their labels y(i) are combined with a delimiter token in between to create a training sequence S(i), which is required and suitable for the next token prediction task. This method ensures that the model itself learns how to generate natural and coherent sentences from structured meaning representations. Each training example is processed independently, allowing the model to learn the semantic relationship between meaning representations and their corresponding sentences, thereby enhancing the quality of text generation.

### 3.3 DoRA integration and weight decomposition

Traditional LoRA adapters exhibit inherent limitations in their learning patterns that constrain the expressiveness of weight updates during fine-tuning. Recent weight decomposition analysis has revealed that LoRA demonstrates a positive correlation between magnitude and directional changes, forcing proportional updates to both components simultaneously. This creates an issue, as it has been noticed that in full fine-tuning, there is a negative correlation between the magnitude and directional updates, which means significant changes can happen to either of the two components with minimal changes in the other, allowing for far better adaptation patterns. With the development of weight-decomposed low-rank adaptation (DoRA), these fundamental limitations are being resolved. DoRA represents one of the latest breakthroughs in PEFT techniques, employed in our work to achieve learning patterns that more closely resemble full fine-tuning while maintaining the efficiency benefits of low-rank adaptation.

To achieve similar results, as seen in full fine-tuning, DoRA decomposes the pre-trained weights into magnitude and directional components. The magnitude component and directional components are then updated separately, as discussed ahead.

Initially, the weight matrix is decomposed as shown in Equation 4, where $W\in {\mathbb{R}}^{d\times k}$ :


$$W=m*(V/V_{c})=W_{c}*(\frac{W}{{||W||}_{c}})\;$$ (4)

where $m\in {\mathbb{R}}^{1\times k}$ represents the magnitude vector, $V\in {\mathbb{R}}^{d\times k}$ represents the directional matrix, and ‖⋅‖c denotes the column-wise norm. This decomposition ensures that each column of V/||V||_c remains a unit vector, while the corresponding scalar in m defines the magnitude of each vector.

DoRA then applies the following fine-tuning formulation as shown in Equation 5:


$$W^{\prime }=m\;\frac{\left(W_{o}+\mathrm{BA}\right)}{{||W_{o}+\mathrm{BA}||}_{c}}\;$$ (5)

where m is the trainable magnitude vector initialized as $Wo_{c}$ , $W_{0}$ represents the frozen pre-trained weight matrix, and BA indicates the low-rank directional update applied through LoRA to the directional component.

As discussed, DoRA maintains the same effect as full fine-tuning by allowing the updates of magnitude to stay independent of the directional updates and vice versa via the dual component training strategy. The directional component is updated using LoRA, enabling low-rank adaptations to the directions, while the magnitude component is directly optimized as a learnable vector, allowing for control over the scale of the weight column. This decomposition allows the model to make better adjustments according to the data by permitting substantial changes to either of the two components, which is something that LoRA struggles to achieve due to the coupling constraint it faces.

### 3.4 U-shaped architecture design

If taken into the context of distributed training and client privacy, the traditional split learning approach, which places only initial layers on clients, treats each model component independently and does not preserve the privacy of final predictions. Losing output privacy would impact the framework’s applicability in understanding sensitive text generation scenarios. The U-shaped architecture easily addresses this issue by ensuring that both the initial and final processing of human-readable text or tokens, which can be decoded into text, stays on the client device throughout the process of fine-tuning, while the intermediate heavy computations can be done on the server side to reduce the load on the client. This U-shaped configuration ensures the same privacy that can be achieved with fully locally hosted fine-tuning, allowing the client to maintain control over both the input data and output predictions.

**Figure 4:**
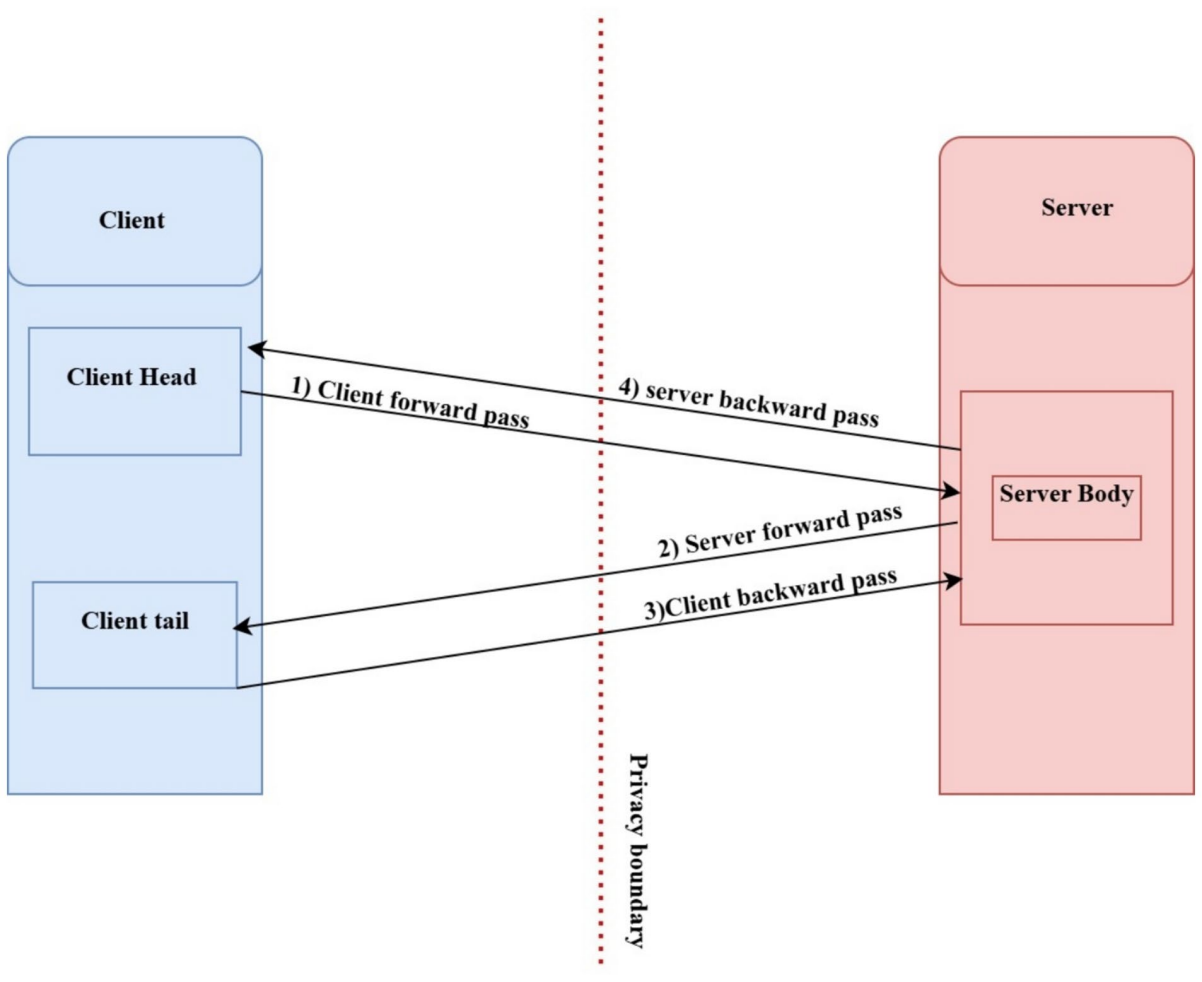
U-shaped split learning framework.

Traditional model splitting used in split learning frameworks, such as linear layer partitioning, introduces a deterministic and fixed pattern based on simple layer boundaries to provide computational distribution. The linear splitting assigns each layer to either the client or server based on predetermined boundaries using computational load balancing at varying granularities. The alternating client–server assignments distribute the computational load in a way that allows the framework to balance privacy and efficiency while maintaining communication overhead (Figure 4).

From a privacy-by-design perspective, the U-shaped partition provides a structural guarantee: the server never receives raw input tokens or final output logits, only intermediate hidden-state activations A_h and A_s , which are high-dimensional floating-point tensors without direct semantic interpretability. This architectural isolation is qualitatively stronger than the conventional linear split, where server-side layers include the output projection and therefore see probability distributions over the vocabulary, from which labels can, in principle, be inferred. The current work does not claim differential privacy or information-theoretic leakage bounds; rather, it establishes a design that is inherently more resilient to label-inference attacks than linear-split alternatives. Formal privacy analysis using frameworks such as mutual information leakage bounds [cite Reviewer 1’s suggested references] is identified as future work.

### 3.5 Communication protocols

The proposed framework also implements a distributed communication protocol that controls the synchronous training process across the three components of the network while preserving the privacy boundary and maintaining minimal overhead. The model is partitioned into a client head (H), server body (S) and a client tail (T), where each component maintains independent parameter optimization through dedicated AdamW optimizers and synchronized schedulers. The entire training process happens through multiple tensor exchanges both during the forward pass for activation and cache propagation and during the backward pass for gradient flow.

#### 3.5.1 Forward propagation protocol

The forward pass initiates the entire process of computation and gradient flow across the split. Given the input configuration where x represents token identifiers, M denotes the expanded causal-padding mask, and K encompasses concatenated past key-values from previous decoding steps, the forward propagation unfolds as follows:

1.
A_h,K_h=H(x,M,use_cache=True)

2.
A_s,K_s=S(A_h,M,K_h)

3.
logits=T(A_s,M,K_s)

where A_h is the hidden state representation, K_h is the client-side key-value cache, and K_s is the server-side key-value cache.

##### 3.5.1.1 Client head processing

The head component tokenizes the text and converts it to embeddings, followed by positional encoding integration. Then, the input sequence is processed through the initial L_h transformer blocks, which generate the hidden state representations A_h and maintain a key-value cache K_h , which are then transmitted to the server. This ensures that raw input tokens never traverse network boundaries while enabling downstream processing.

##### 3.5.1.2 Server body computation

The server receives the head activations along with the attention mask and cached key-value pairs, then propagates these representations through the intermediate L_s transformer blocks. The server also adds its own cache K_s , which is necessary to maintain autoregressive consistency, and then returns the activations A_s back to the client to its tail component. All of these computations on the server side operate exclusively on the basis of abstract feature representation, which preserves privacy.

##### 3.5.1.3 Client tail finalization

The server-side received activations are received by the tail component of the client, which then completes the final computation through the remaining transformer blocks and then applies layer normalization to produce the final output. This design ensures that the input processing and the output generation remain on the client side.

The protocol maintains computational efficiency by streaming all past key-values forward throughout the pipeline, enabling autoregressive text generation to require only a single round-trip communication per generated token during inference.

#### 3.5.2 Backward propagation protocol

The backward pass implements a precise gradient flow mechanism that reverses the forward computation path while maintaining distributed parameter optimization. Following cross-entropy loss computation at the tail client, gradient propagation proceeds as δoutput→δA_s→δA_h→δ embeddings.

##### 3.5.2.1 Gradient flow orchestration

The gradients flow in the reverse order of activations through the T→S→H path, where each split executes its own backward operations, applies gradient clipping for numerical stability, and performs optimizer steps independently. This design ensures that no gradient information crosses network boundaries while maintaining full backpropagation connectivity.

##### 3.5.2.2 Distributed parameter updates

Each component utilizes its own AdamW optimizer to manage its parameter optimization, which maintains stability while learning. The client tail initiates the gradient computation; then the server propagates gradients through body layers while updating its own parameters, and the flow ends at the client head while updating the very first layers of the architecture.

#### 3.5.3 Optimizer and scheduler coordination

##### 3.5.3.1 Independent optimization

All the splits have their own AdamW optimizer, as mentioned earlier, which allows for components’ specific learning rate adaptation while also ensuring synchronized convergence of the entire network. This prevents bottlenecks and enables parallel parameter updates.

##### 3.5.3.2 Linear scheduling protocol

A linear learning rate scheduler with 500 warm-up steps is attached to every optimizer. The schedule implements a linear increase from zero to the base learning rate during the initial 500 training steps, followed by linear decay to zero over the remaining training duration.

#### 3.5.4 Robustness and reliability measures

##### 3.5.4.1 Gradient stability

The protocol implements gradient clipping across every architectural split to prevent the issue of gradient explosion and ensure numeric stability. Additionally, checks are performed on the output, losses, and gradients for signs of numeric instability, with the mini-batches being automatically skipped if they fail the following checks to maintain training continuity without interruptions.

The workflow for building and training the proposed framework is demonstrated in Algorithm 2.

ALGORITHM 2 U-splitDoRA model training
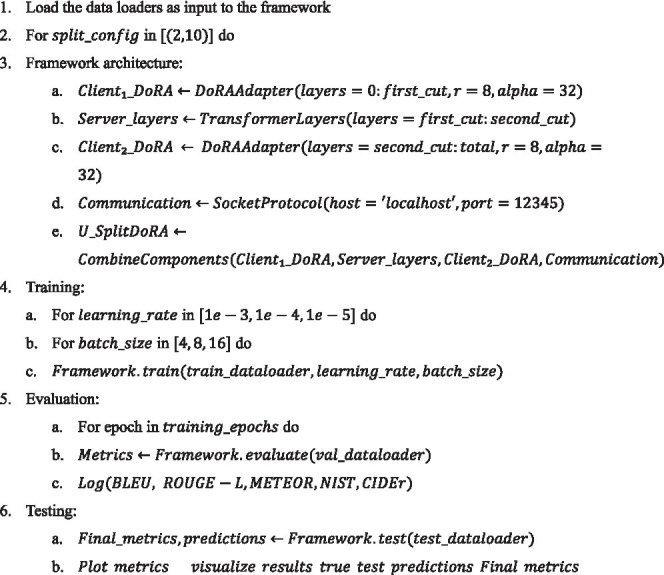

Regarding communication overhead, in the current U-shaped setup, each forward pass requires two activation transfers (client-head to server, and server to client-tail) per training step, and two gradient transfers in the backward pass. The communication volume is O(batch_size×hidden_dim×seq_len) per transfer, identical to SplitLoRA’s forward pass communication. The U-shaped design does not introduce additional communication rounds compared to a linear split; it only relocates the output layers to the client. Quantitative end-to-end communication benchmarking under realistic network conditions is identified as future work.

## 4 Experiments and results

The experimental setup, dataset details, model, and metrics used for experimentation are provided in this section. Further, the training performance results of the proposed U-SplitDoRA framework are reported and discussed.

### 4.1 Experimental setup

#### 4.1.1 Hardware and software

The U-SplitDoRA framework is built using Python 3.11 and PyTorch 1.7.1. The model is trained on NVIDIA A100 GPUs.

#### 4.1.2 Dataset

The E2E NLG dataset (Novikova et al., 2017) is adopted as the evaluation benchmark. It is one of the most widely used limited-domain NLG benchmarks, having served as the basis for the E2E NLG Challenge. Importantly, the E2E dataset is the established benchmark for the SL + PEFT paradigm: SplitLoRA (Lin et al., 2024) and HsplitLoRA (Lin et al., 2025), the direct antecedents of this work, both evaluate on E2E with GPT-2 models, making it the natural and methodologically required choice for enabling direct performance comparison. The number of training, validation, and test samples are 42,000, 4,600, and 4,600, respectively.

#### 4.1.3 Models

To implement the proposed U-SplitDoRA framework, two large language models are used, GPT-2-S and GPT-2-M (Radford et al., 2019), where GPT-2-S is a pre-trained model with 12 layers of transformer blocks and over 117 million parameters, and GPT-2-M has 24 layers and 345 million parameters.

#### 4.1.4 Hyperparameters and their value settings

The hyperparameter values set to conduct experiments are given in Table 1.

**Table 1:** Hyperparameters and their value.

Hyperparameter	Value
Batch size	8
Learning rate	0.0002
LoRA α	32
Optimizer	AdamW
Sequence length	256
Ranks	4, 8
Epochs	3
Warmup steps	500
Target_layers	“c_attn,” “c_proj,” “c_fc”

This study considers a single-client configuration. Since only one client is present, no federated aggregation step is performed. The entire E2E dataset is held at the single client. As a result, data partitioning strategy, IID/non-IID characterization, and aggregation frequency are not applicable parameters in this configuration. The cuts for GPT-2-S are made at layers 2 and 10, providing 2 layers to the head, 2 to the tail, and 8 to the body. For GPT-2-M, the same ratio of layers is split between the head, body, and tail. Communication latency was not explicitly modeled as a variable; the framework focuses on accuracy and convergence performance, with latency analysis identified as future work. All baseline frameworks (CentLoRA, SplitLoRA, FedIT) were evaluated on the same E2E dataset with identical preprocessing steps and the same rank settings (*r* = 4, *r* = 8) to ensure a fair comparison.

#### 4.1.5 Evaluation metrics

The five evaluation metrics are chosen to match the standard E2E NLG Challenge evaluation protocol (Novikova et al., 2017), enabling direct comparison with all prior work. BLEU (Papineni et al., 2002) and NIST (Doddington, 2002) measure n-gram precision; METEOR (Lavie and Agarwal, 2007) incorporates stemming and synonym matching for recall-aware evaluation; ROUGE-L (Chin-Yew, 2004) captures the longest common subsequence structure; and CIDEr (Vedantam et al., 2015) weights n-gram matches by their informativeness using TF-IDF. Together, these metrics provide complementary views of generation quality.

#### 4.1.6 SOTA frameworks for comparison studies

Extensive performance evaluations of U-SplitDoRA are conducted, and the results are compared with state-of-the-art LLM fine-tuning frameworks. This includes:

- Centralized low-rank adaptation (CentLoRA; Hu et al., 2022)—LLMs’ LoRA adapters are updated at a centralized server.
- Split low-rank adaptation (SplitLoRA; Lin et al., 2024)—A split learning paradigm is implemented, which partitions the LLM fine-tuning at the server and client sides. The LoRA adapters at the client end are aggregated at regular intervals.
- Federated Instruction Tuning (FedIT; Zhang et al., 2023)—The client-side server fine-tunes the full LLM model locally, and LoRA adapters are aggregated at the central server.

To ensure a fair comparison, all frameworks were trained on the same E2E NLG dataset under the same 60–20-20 split, identical preprocessing pipeline, the same sequence length (256), and the same LoRA rank settings (*r* = 4 and *r* = 8). CentLoRA and SplitLoRA use LoRA as the PEFT method on GPT-2-S and GPT-2-M; FedIT uses LoRA-based parameter aggregation. U-SplitDoRA uses DoRA under the same rank budget. No additional tuning budget was allocated to U-SplitDoRA beyond what was made available to the baselines.

### 4.2 Results and discussions

The performance results of the proposed U-SplitDoRA are presented in this section, evaluating the robustness of the proposed framework.

#### 4.2.1 Converged accuracy

**Figure 5:**
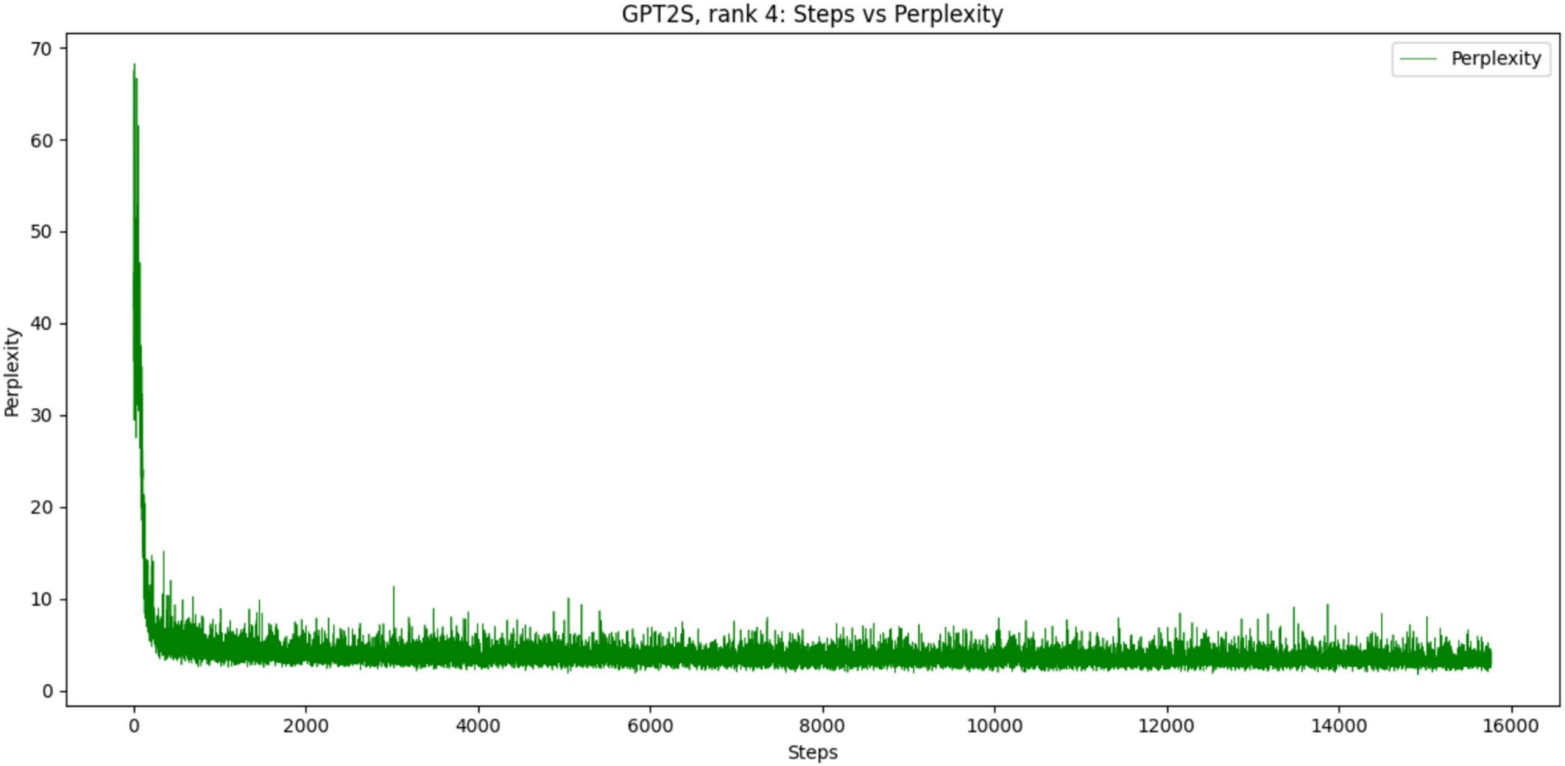
U-SplitDoRA training performance convergence on GPT-2-S (See Tables 2, 3 for the corresponding converged metric values).

The performance of U-SplitDoRA while training is presented in Figure 5. The converged accuracy is measured using perplexity (PPL) as a performance evaluation metric and is used to assess the models’ prediction ability. A lower PPL score indicates better prediction by the model. U-SplitDoRA attains higher accuracy and a faster convergence rate. Figure 5 shows the convergence of the U-SplitDoRA framework, trained on the GPT-2-S model with the rank = 4 setting.

**Figure 6:**
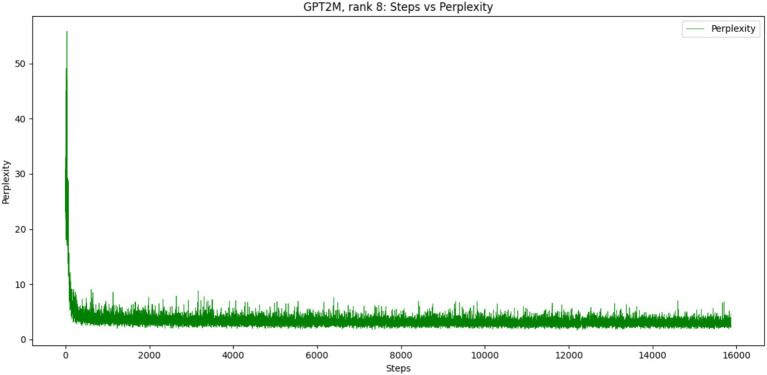
U-SplitDoRA training performance convergence on GPT-2-M (See Tables 2, 3 for the corresponding converged metric values).

Figure 6 shows the convergence of the U-SplitDoRA framework, trained on the GPT-2-M model with the rank = 8 setting.

**Figure 7:**
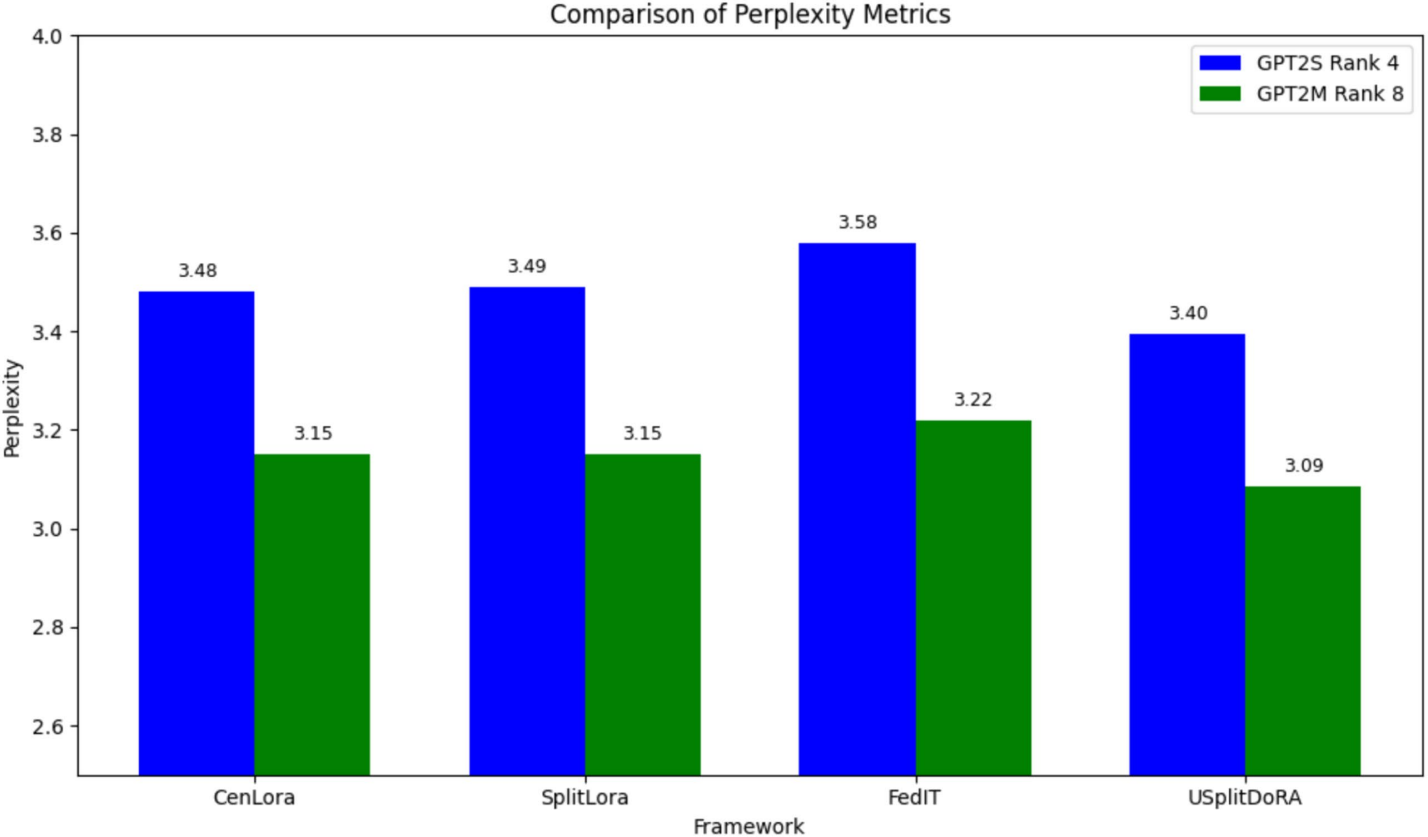
Comparison of converged accuracy on GPT-2-S and GPT-2-M (See Tables 2, 3 for the corresponding converged metric values).

The training performance of the proposed U-SplitDoRA framework, in comparison to other SOTA methods, is shown in Figure 7 for the GPT-2-S and GPT-2-M models. U-SplitDoRA attains the best PPL and outperforms CentLoRA, SplitLoRA, and FedIT.

The robustness and adaptability are rooted in U-SplitDoRA’s U-shaped split learning paradigm to preserve data privacy and DoRA as a PEFT method for better learning capacity. U-SplitDoRA works by offloading the major training task on the server end, like SplitLoRA and FedIT, with only a lesser task of LLM trained on the client end, but improved performance is observed due to the novel integration of DoRA as a PEFT method in place of LoRA. The performance exhibited by CentLoRA is still on par, but it suffers from scalability issues and is not suitable for a distributed learning environment on a larger scale.

An extensive performance evaluation using other performance metrics is conducted, and results are presented in Tables 2, 3. It is evident that U-SplitDoRA attained better scores across the metrics experimented with using the E2E dataset compared to the other fine-tuning frameworks.

**Table 2:** Performance comparison on GPT-2-S using different metrics on the E2E NLG Dataset.

Model	Fine-tuning framework	BLEU	NIST	METEOR	ROUGE-L	CIDEr
GPT-2-S (*r* = 4)	CentLoRA	69.41	8.7824	0.4610	70.70	2.4713
	SplitLoRA	68.79	8.7259	0.4572	69.84	2.4411
	FedIT	67.73	8.6148	0.4494	68.59	2.3817
	**U-SplitDoRA** **(Proposed)**	**69.52**	**8.7921**	**0.4812**	**71.01**	**2.4912**
GPT-2-S (*r* = 8)	CentLoRA	69.37	8.7735	0.4624	70.96	2.4572
	SplitLoRA	68.76	8.6931	0.4588	70.17	2.4165
	FedIT	68.39	8.6745	0.4590	70.24	2.4450
	**U-SplitDoRA** **(Proposed)**	**69.82**	**8.7917**	**0.4819**	**71.05**	**2.4697**

Best scores are in bold.

**Table 3:** Performance comparison on GPT-2-M using different metrics on the E2E NLG Dataset.

Model	Fine-tuning framework	BLEU	NIST	METEOR	ROUGE-L	CIDEr
GPT-2-M (*r* = 4)	CentLoRA	69.78	8.7820	0.4667	71.62	2.5301
	SplitLoRA	70.09	8.8075	0.4667	71.60	2.5370
	FedIT	69.78	8.7836	0.4642	**71.87**	2.4819
	**U-SplitDoRA** **(Proposed)**	**71.28**	**8.8096**	**0.4734**	71.73	**2.5400**
GPT-2-M (*r* = 8)	CentLoRA	70.57	8.8557	0.4688	72.17	2.5405
	SplitLoRA	69.18	8.7189	0.4631	71.30	2.5156
	FedIT	69.55	8.7358	0.4661	71.46	2.4980
	**U-SplitDoRA** **(Proposed)**	**70.58**	**8.8624**	**0.4793**	**72.89**	**2.5462**

Best scores are in bold.

A closer inspection of the results reveals nuances worth noting: for GPT-2-M at rank 4, FedIT achieves a marginally higher ROUGE-L (71.87) compared to U-SplitDoRA (71.73), and for GPT-2-M at rank 8, CentLoRA’s BLEU (70.57) and U-SplitDoRA’s BLEU (70.58) are effectively tied. These cases indicate that the performance advantages of U-SplitDoRA are consistent but not always dominant on every individual metric. The broader advantage of U-SplitDoRA lies in the combination of competitive accuracy, faster convergence, and superior privacy-by-design architecture—characteristics that existing baselines do not offer simultaneously.

**Figure 8:**
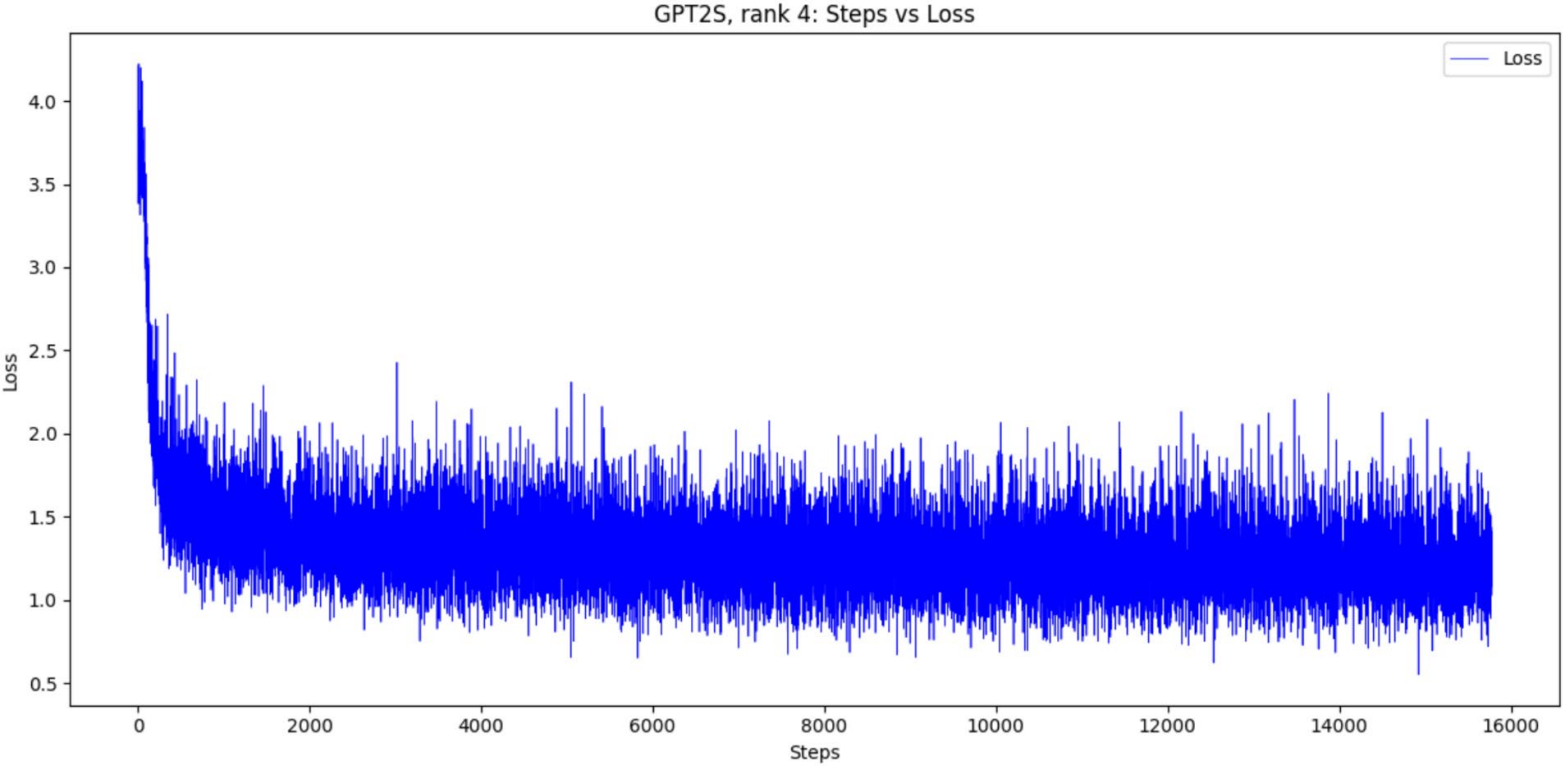
Training loss for GPT-2-S model.

**Figure 9:**
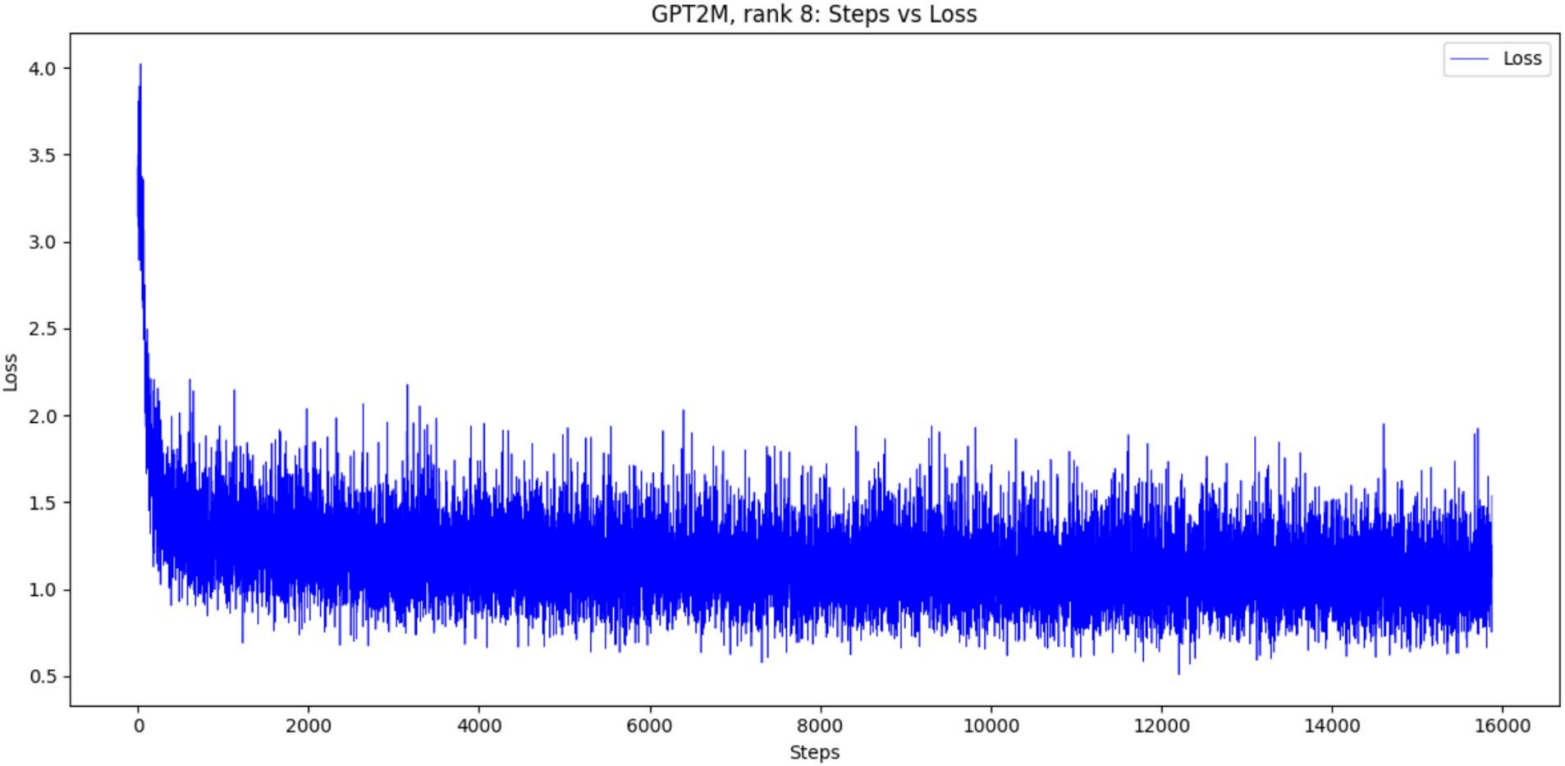
Training loss for GPT-2-M model.

Figures 8, 9 show loss along various steps during training for the GPT-2-S and GPT-2-M models, respectively.

#### 4.2.2 Converged time

**Figure 10:**
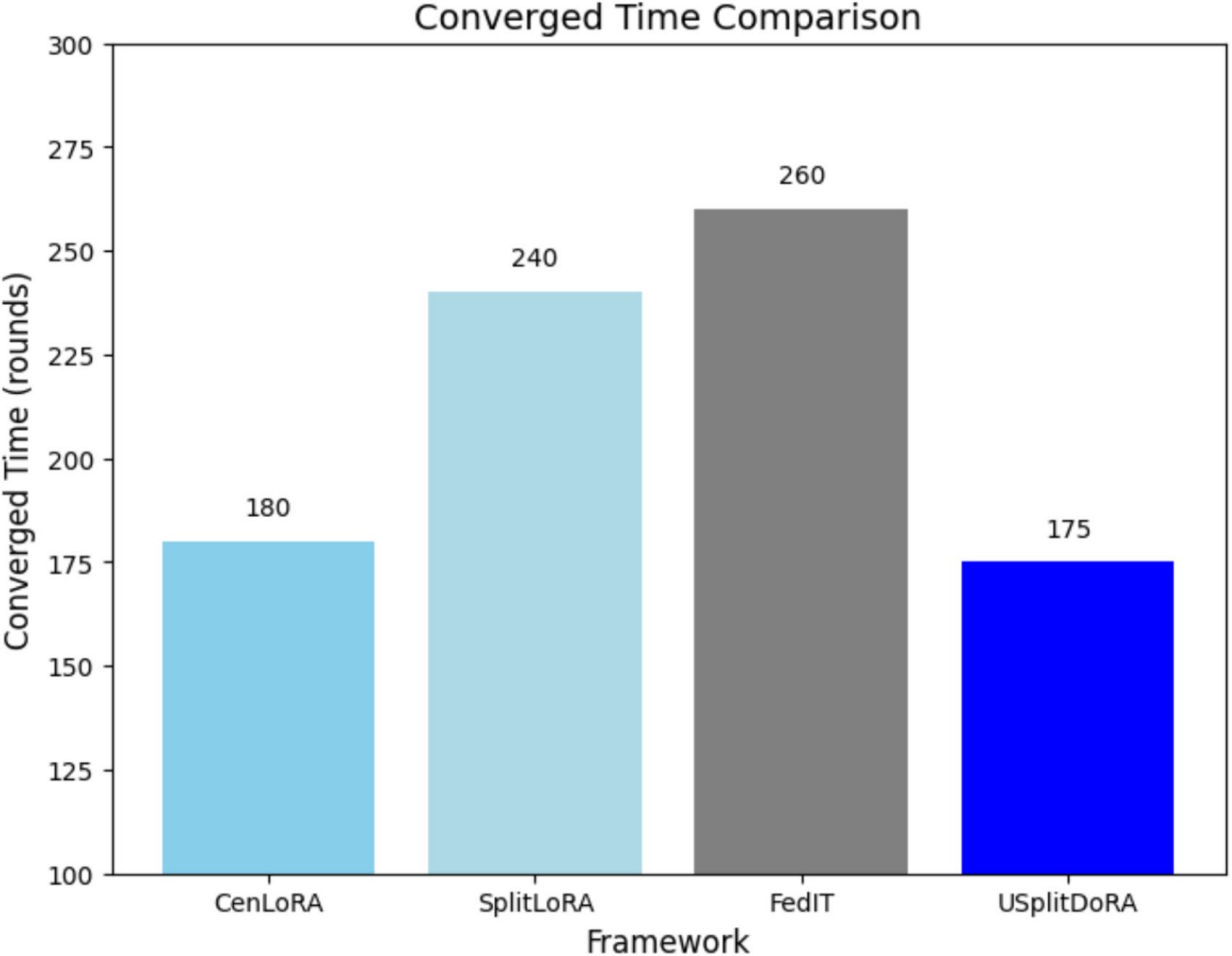
Comparison of converged time on GPT-2-M.

The convergence rate of U-SplitDoRA and other benchmarks is shown in Figure 10 for GPT-2-M. It can be observed that U-SplitDoRA converges faster than CentLoRA, SplitLoRA, and FedIT. All the parallel training-based frameworks perform better than CentLoRA. U-SplitDoRA converges faster, and this gain is attributed to DoRA as a PEFT method, which works through weight decomposition to learn and adapt better. SplitLoRA’s convergence performance is on par with U-SplitDoRA.

#### 4.2.3 Ablation studies

To further assess the contribution of the proposed LLM fine-tuning framework “U-SplitDoRA,” we perform comprehensive ablation studies. Each variant is designed by selectively excluding or altering specific modules, allowing us to analyze their individual impact on overall performance. The variants include (i) linear split + DoRA vs. U-split + DoRA (Proposed) to measure the privacy-architecture contribution, and (ii) U-split + LoRA vs. U-split + DoRA (Proposed) to measure the PEFT contribution independently. The attained results for (*r* = 8) are provided in Table 4.

**Table 4:** Ablation study results on the E2E NLG dataset.

Model	Fine-tuning framework	BLEU	NIST	METEOR	ROUGE-L	CIDEr
GPT-2-S (*r* = 8)	linear split + DoRA	69.11	8.7344	0.4792	70.97	2.4592
	**U-SplitDoRA** **(Proposed)**	**69.82**	**8.7917**	**0.4819**	**71.05**	**2.4697**
	U-split + LoRA	69.23	8.7128	0.4781	70.94	2.4523
	**U-SplitDoRA** **(Proposed)**	**69.82**	**8.7917**	**0.4819**	**71.05**	**2.4697**
GPT-2-M (*r* = 8)	linear split + DoRA	69.88	8.7811	0.4672	72.01	2.5261
	**U-SplitDoRA** **(Proposed)**	**70.58**	**8.8624**	**0.4793**	**72.89**	**2.5462**
	U-split + LoRA	69.82	8.7534	0.4611	71.90	2.5216
	**U-SplitDoRA** **(Proposed)**	**70.58**	**8.8624**	**0.4793**	**72.89**	**2.5462**

Best scores are in bold.

The results for the linear split + DoRA vs. U-split + DoRA (Proposed) studies show that, across both GPT-2-S and GPT-2-M, the proposed U-Split + DoRA consistently outperforms the linear split configuration. This demonstrates that the architecture itself contributes significantly, beyond just privacy preservation.

The results for U-split + LoRA vs. U-split + DoRA (Proposed) show that the DoRA-based model consistently achieves superior performance across all metrics. DoRA provides more stable adaptation by decoupling direction and magnitude updates.

Thus, when integrating both contributions (U-shaped split + DoRA), the proposed U-SplitDoRA achieves the best performance across all configurations, indicating a complementary effect.

## 5 Conclusion and future work

In this work, we present a privacy-preserved split learning-based LLM fine-tuning framework called U-SplitDoRA. It is constructed on a split federated learning framework combining the benefits of parallel training and model splitting, make it a privacy-preserved and collaborative learning framework. A U-shaped server–client paradigm is suggested for split learning to improve security and preserve the privacy of client data. Weight-decomposed low-rank adaptation (DoRA) is used as a PEFT technique that exhibits generalization and more reliable training through controlled adaptation by updating the directional component via LoRA while independently optimizing the magnitude as a learnable scalar vector. The performance of the U-SplitDoRA framework is validated extensively, and it converges and attains the target accuracy in competitive timing compared to other state-of-the-art centralized and split learning-based LLM fine-tuning frameworks. This shows the robustness and significance of U-SplitDoRA. The future research direction is to design the model by applying quantization techniques to make it more efficient.

## Data Availability

The original contributions presented in the study are included in the article/supplementary material, further inquiries can be directed to the corresponding author.

## References

[ref1] Anil R. (2023). PaLM 2 technical report. arXiv preprint arXiv 2305:10403.

[ref2] Anthropic, The Claude 3 model family: opus, sonnet, haiku, https://www.anthropic.com/news/claude-3-family, (2023) (accessed 4 April 2025).

[ref3] Basyal L. Sanghvi M. (2023). Text summarization using large language models: a comparative study of MPT-7b-instruct, falcon-7b-instruct, and OpenAI chat-GPT models. arXiv preprint arXiv 2310:10449.

[ref4] Cai D. Wu Y. Wang S. Lin F. Xiaozhu Xu M., “Efficient federated learning for modern NLP,” in: *Proceedings of the 29th Annual International Conference on Mobile Computing and Networking (ACM MobiCom '23)*, (2023), pp. 1–16.

[ref5] Chin-Yew L., “ROUGE: a package for automatic evaluation of summaries,” in: *Text Summarization branches out: Proceedings of the ACL- 04 Workshop*, (2004), pp. 74–81.

[ref6] DeepSeek-AI Guo D. Yang D. Zhang H. (2025). DeepSeek-R1: incentivizing reasoning capability in LLMs via reinforcement learning. arXiv preprint.

[ref7] DeepSeek-AI Liu A. (2024). DeepSeek-V3 technical report. arXiv preprint, 2412:19437.

[ref8] Devlin J. Chang M.-W. Lee K. Toutanova K. “Bert: pre-training of deep bidirectional transformers for language understanding,” in: *Proceedings of the 2019 Conference of the North American Chapter of the Association for Computational Linguistics: Human Language Technologies (NAACL-HLT)*, (2019), pp. 4171–4186.

[ref9] Doddington G., “Automatic evaluation of machine translation quality using n-gram co-occurrence statistics,” in*: Proceedings of the Second International Conference on Human Language Technology Research*, (2002), pp. 138–145.

[ref10] Driess D., “PaLM-E: an embodied multimodal language model,” in: *Proceedings of the 40th International Conference on Machine Learning (ICML'23)*, (2023), pp. 8469–8488.

[ref11] Duan Q. Hu S. Deng R. Lu Z. (2022). Combined federated and Split learning in edge computing for ubiquitous intelligence in internet of things: state-of-the-art and future directions. Sensors 22, 1–37. doi: 10.3390/s22165983, 36015747 PMC9414384

[ref12] Fan T. Kang Y. Ma G. Chen W. Wei W. Fan L. . (2023). Fate-LLM: a industrial grade federated learning framework for large language models. arXiv preprint arXiv 2310:10049.

[ref13] Gemini Team (2024). Gemini 1.5: unlocking multimodal understanding across millions of tokens of context. arXiv e-prints, arXiv 2403:05530.

[ref14] Hamadi R. (2023). Large language models meet computer vision: a brief survey. arXiv preprint arXiv.

[ref15] He J. Zhou C. Ma X. Berg-Kirkpatrick T. Neubig G. *Proceedings of the 10th International Conference on Learning Representations (ICLR-2022)* (2022)

[ref16] Hou X. Zhao Y. Liu Y. Yang Z. Wang K. Li L. . (2024). Large language models for software engineering: a systematic literature review. ACM Trans. Softw. Eng. Methodol. arXiv preprint, 33:220.

[ref17] Houlsby N. “Parameter-efficient transfer learning for NLP,” in: *Proceedings of the 36^th^ International Conference on Machine Learning*, (2019).

[ref18] Hu E. J. Shen Y. Wallis P., “LoRA: low-rank adaptation of large language models,” in: *Proceedings International Conference on Learning Representations*, (2022).

[ref19] Huang Y. Liu W. (2024). Evaluating the translation performance of large language models based on Euas-20. arXiv preprint arXiv 2408:03119.

[ref20] Hyeon-Woo N. Ye-Bin M. Tae-Hyun O. “FedPara: low-rank Hadamard product for communication-efficient federated learning,” *International Conference on Learning Representations*, (2022).

[ref21] Koneˇcn’y J. McMahan H. B. Yu F. X. Richt’arik P. Suresh A. T. Bacon D. (2016). Federated learning: strategies for improving communication efficiency. arXiv preprint arXiv.

[ref22] Kopiczko D. J. Blankevoort T. Asano Y. M., “VeRA: vector-based random matrix adaptation,” *International Conference on Learning Representations*, (2024).

[ref23] Kuang W. Qian B. Li Z. Chen D. Gao D. Pan X. . (2023). FederatedScope-LLM: a comprehensive package for fine-tuning large language models in federated learning. arXiv preprint arXiv.

[ref24] Lavie A. Agarwal A., “METEOR: an automatic metric for MT evaluation with high levels of correlation with human judgments,” in: *Proceedings of the Second Workshop on Statistical Machine Translation. Association for Computational Linguistics*, (2007), pp. 228–231.

[ref25] Lester B. Al-Rfou R. Constant N., “The power of scale for parameter-efficient prompt tuning,” in *Proceedings of the 2021 Conference on Empirical Methods in Natural Language Processing*, (2021), pp. 3045–3059.

[ref26] Liang X. Wang H. Controllable text generation for large language models: a survey, Available online at: https://arxiv.org/abs/2408.12599 (Accessed May 10, 2025).

[ref27] Lin Z. Hu X. Zhang Y. Chen Z. Fang Z. Chen X. . (2024). SplitLoRA: a split parameter-efficient fine-tuning framework for large language models. arXiv preprint arXiv 2407:00952.

[ref28] Lin Z. Qu G. Chen X. Huang K. (2024). Split learning in 6G edge networks. IEEE Wirel. Commun. 31, 170–176. doi: 10.1109/MWC.014.2300319

[ref29] Lin Z. Zhang Y. Chen Z. Fang Z. Chen X. Vepakomma P. . (2025). HSplitLoRA: a heterogeneous split parameter-efficient fine-tuning framework for large language models. arXiv preprint arXiv.

[ref30] Lin Z. Zhu G. Deng Y. Chen X. Gao Y. Huang K. . (2024). Efficient parallel split learning over resource-constrained wireless edge networks. IEEE Trans. Mob. Comput. arXiv preprint. 23, 9224–9239. doi: 10.1109/TMC.2024.3359040

[ref31] Liu S. Chien-Yi W. Hongxu Y., “DoRA: weight-decomposed low-rank adaptation”, in: *Proceedings of the 41st International Conference on Machine Learning (ICML'24)*, (2024), pp. 32100–32121.

[ref32] Liu Y. Shi K. “On learning to summarize with large language models as references,” in *Proceedings of NAACL 2024*, (2025).

[ref33] Lyu S. Lin Z. Qu G. Chen X. Huang X. Li P. (2023). Optimal resource allocation for U-shaped parallel split learning. arXiv preprint arXiv.

[ref34] Mahabadi R. K. Ruder S. Dehghani M. Henderson J., “Parameter-efficient multi-task fine-tuning for transformers via shared Hypernetworks,” in: *Proceedings of the 59^th^ Annual Meeting of the Association for Computational Linguistics and the 11^th^ International Joint Conference on Natural Language Processing*, (2021), pp. 565–576.

[ref35] Mark C. (2021). Evaluating large language models trained on code. arXiv preprint.

[ref36] McMahan B. Moore E. Ramage D. Hampson S. Arcas B. A., “Communication-efficient learning of deep networks from decentralized data,” in: *Proceedings of the 20th International Conference on Artificial Intelligence and Statistics*, (2017), pp. 1273–1282.

[ref37] Meng X. Yan X. Zhang K. Liu D. Cui X. Yang Y. . (2024). The application of large language models in medicine: a scoping review. iScience 27:109713. doi: 10.1016/j.isci.2024.109713, 38746668 PMC11091685

[ref38] Novikova J. Duˇsek O. Rieser V., “The E2E dataset: new challenges for end-to-end generation,” in *Proceedings of the 18th Annual SIGdial Meeting on Discourse and Dialogue*, (2017), pp. 201–206.

[ref39] OpenAI (2023). GPT-4 technical report. arXiv preprint arXiv 2303:08774.

[ref40] Papineni K. Roukos S. Ward T. Zhu W., “BLEU: a method for automatic evaluation of machine translation,” in: *Proceedings of the 40th Annual Meeting of the Association for Computational Linguistics, Association for Computational Linguistics*, (2002), pp. 311–318.

[ref41] Radford A. Wu J. Child R. Luan D. Amodei D. Sutskever I. . (2019). Language models are unsupervised multitask learners. OpenAI Blog. arXiv preprint.

[ref42] Razdaibiedina A. Mao Y. Khabsa M. Lewis M. Huo R., “Residual prompt tuning: improving prompt tuning with residual Reparameterization,” in: *Findings of the Association for Computational Linguistics: ACL 2023*, (2023), pp. 6740–6757.

[ref43] Ryu J. Won D. Lee Y., “A study of Split learning model,” in: *16th International Conference on Ubiquitous Information Management and Communication (IMCOM)*, (2022), pp. 1–4.

[ref44] Shijie W. Ozan I. (2023). BloombergGPT: a large language model for finance. arXiv preprint arXiv 2303:17564.

[ref45] Singhal K. Tu T. Gottweis J. Sayres R. Wulczyn E. Hou L. . (2025). Towards expert-level medical question answering with large language models. Nat. Med. 31, 943–950. doi: 10.1038/s41591-024-03423-7, 39779926 PMC11922739

[ref46] Thapa C. Mahawaga Arachchige P. C. Camtepe S. Sun L., “SplitFed: when federated learning meets Split learning,” in: *Proceedings of the AAAI Conference on Artificial Intelligence*, (2022), pp. 8485–8493.

[ref47] Touvron H. Lavril T. Izacard G. Martinet X. Lachaux M.-A. Lacroix T. . (2023). LLaMA: open and efficient foundation language models. arXiv preprint arXiv.

[ref48] Vaswani A. Shazeer N. Parmar N., “Attention is all you need,” in: *Proceedings of the 31st International Conference on Neural Information Processing Systems (NIPS'17)*, (2017), pp. 6000–6010.

[ref49] Vavekanand R. (2026). A comprehensive review of multimodal large language models for medical imaging and omics data. Arch. Computat. Methods Eng. doi: 10.1007/s11831-026-10504-y

[ref50] Vavekanand R. Ali Laghari A. Kumar T. (2026). Applications and limitations of large language models to integrate medical context: a comprehensive review. Iran J. Comput. Sci. arXiv preprint. 9. doi: 10.1007/s42044-025-00360-7

[ref51] Vedantam R. Zitnick C. Lawrence Parikh D., “CIDEr: Consensusbased image description evaluation,” in: *Proceedings of the 2015 IEEE Conference on Computer Vision and Pattern Recognition (CVPR)*, (2015), pp. 4566–4575.

[ref52] Vepakomma P. Gupta O. Swedish T. Raskar R. (2018). Split learning for health: distributed deep learning without sharing raw patient data. arXiv preprint arXiv.

[ref53] Villalobos P. Sevilla J. Heim L. Besiroglu T. Hobbhahn M. Ho A. (2022). Will we run out of data? An analysis of the limits of scaling datasets in machine learning. arXiv preprint arXiv.

[ref54] Wang Y. (2023). Non-intrusive adaptation: input-centric parameter-efficient fine-tuning for versatile multimodal modeling. arXiv preprint arXiv 2310:12100.

[ref55] Ye R. “OpenFedLLM: training large language models on decentralized private data via federated learning,” in: *Proceedings of the 30th ACM SIGKDD Conference on Knowledge Discovery and Data Mining (KDD '24)*, (2024), pp. 6137–6147.

[ref56] Yue M. (2025). A survey of large language model agents for question answering. arXiv preprint arXiv 2503:19213.

[ref57] Zhang Q. “AdaLoRA: adaptive budget allocation for parameter-efficient fine-tuning,” *The 11^th^ International Conference on Learning Representations (ICLR 2023)*, arXiv preprint. (2023).

[ref58] Zhang Y. Chen H. Lin Z. Chen Z. Zhao J. (2024). FedAC: a adaptive clustered federated learning framework for heterogeneous data. arXiv preprint arXiv.

[ref59] Zhang S. Cheng G. Li Z. Wu W. (2025). SplitLLM: hierarchical split learning for large language model over wireless network. arXiv preprint.

[ref60] Zhang J. Vahidian S. Kuo M. Li C. Zhang R. Wang G. . (2023). Towards building the federated GPT: federated instruction tuning. arXiv preprint arXiv 2305:05644.

[ref61] Zhao Y. Zhang H. Si S. Nan L. Tang X. Cohan A. “Investigating Table-to-text generation capabilities of large language models in real-world information seeking scenarios,” in: *Proceedings of the 2023 Conference on Empirical Methods in Natural Language Processing: Industry Track*, (2023), pp. 160–175.

[ref62] Zhiqiang W. Yiran P. Yanbin L. (2024). Smart expert system: large language models as text classifiers. arXiv preprint. doi: 10.48550/arXiv.2405.10523

[ref63] Zhu W. Liu H. “Multilingual machine translation with large language models: empirical results and analysis,” in: *Findings of the Association for Computational Linguistics: NAACL 2024*. (2024), pp. 2765–2781.

